# Maternal near miss and mortality due to postpartum infection: a cross-sectional analysis from Rwanda

**DOI:** 10.1186/s12884-016-0951-7

**Published:** 2016-07-20

**Authors:** Denis Rwabizi, Stephen Rulisa, Findlater Aidan, Maria Small

**Affiliations:** Department of Obstetrics and Gynecology, University of Rwanda, BP 655 Kigali, Rwanda; Department of Obstetrics and Gynecology, University Teaching Hospital of Kigali, Kigali, Rwanda; University of Western Ontario, London, ON Canada; Department of Obstetrics and Gynecology, Duke University, Durham, NC USA

**Keywords:** Rwanda, Peritonitis, Near miss, Severe maternal morbidity, Maternal mortality

## Abstract

**Background:**

The objective of this study is to evaluate ‘near miss’ and mortality in women with postpartum infections.

**Methods:**

We performed a retrospective review of all patients referred to the University Teaching Hospital of Kigali (CHUK) between January 2012 and December 2013. We identified 117 patients with postpartum infections. Demographic data, length of admission, location of referral, initial surgery and subsequent treatment modalities including antibiotic administration and secondary surgery were recorded. The primary outcome of interest was a composite of maternal mortality and “near miss” defined as more than one laparotomy with/without hysterectomy and prolonged hospitalization.

**Results:**

Diagnoses at CHUK were: pelvic peritonitis (56 %), deep surgical site infection including fasciitis (17 %), and endometritis (15 %). The primary procedures performed prior to transfer were: cesarean section (81 %), septic abortion management (12 %), and vaginal delivery (7 %). Antibiotics were initiated prior to transfer in 66 % of women. Surgery was required in 73 % of patients. Hysterectomies were performed in 22 % of patients. Maternal death occurred in 5 % of the patient population. The primary outcome of severe maternal morbidity and mortality occurred in 90 patients (77 %).

**Conclusion:**

Peritonitis—primarily as a result of cesarean deliveries—is associated with significant morbidity and mortality in our population.

## Background

Millennium Development Goal (MDG) 5 called for a 75 % reduction in Maternal Mortality (MMR) [1] and recent, United Nation Sustainable Development Goals aim for global maternal mortality ratios to reach 70 per 100,000 live births by 2030 [2]. Rwanda achieved substantial gains in maternal mortality reduction. The maternal mortality ratio decreased from an estimated 952/100,000 in the year 2000 to 320/100,000 in 2013 and 210/100,00 in 2014-15 [3]. Continuing this downward trend is a high priority for the Rwanda Ministry of Health. Overall, the cesarean delivery rate is 15 % for the country. The cesarean delivery rate in district hospitals increased from 36 % in 2010 to 45 % in 2011. This increase in cesarean delivery rates is partially attributed to the nation’s reduced maternal and child mortality [4].

Higher cesarean delivery rates, however, may also lead to an increase in maternal morbidity and mortality, as cesarean sections are associated with a 5-fold to 20-fold increased risk of infection compared to vaginal delivery [5]. In Sub-Saharan Africa approximately 9.7 % of maternal mortality is secondary to infection [6].

In 2010, sepsis was the second leading cause of maternal mortality in our center, after post-partum hemorrhage [7].

Previous work from Rwanda identified cesarean delivery related peritonitis as a leading cause of severe maternal morbidity and mortality at the University teaching hospital of Rwanda (CHUK) [8]. CHUK is the largest public hospital in Rwanda and serves as one of the primary teaching and tertiary care referral centers for the country. This study examines factors related to severe morbidity and mortality, or ‘near misses’ in women with postpartum infection.

The World Health Organization applies the term, ‘maternal near miss’ to describe a ‘woman who nearly died but survived a complication that occurred during pregnancy, childbirth or within 42 days of termination of pregnancy’. (8). We evaluate these events at the University teaching hospital of Kigali to both monitor these cases and determine how to prevent or reduce these serious complications [9]. This work focuses on postpartum infectious complications.

## Methods

CHUK is the largest public tertiary hospital in Rwanda and accepts referrals from 29 (over 70 %) district hospitals. The hospital has approximately 3000 admissions and 2000 deliveries annually. The majority of deliveries and transfers from district hospitals are for high risk, complicated pregnancies. The majority of transfers (approximately 50 %) are from the eastern region of the nation. The CHUK admission registry was used to identify patients admitted between January 2012 and December 2013 with a diagnosis of postpartum infection. Admission diagnoses were confirmed by chart review. Obstetrical patients transferred with fever, pelvic pain, severe wound infection, or acute abdomen were included in the study. The CHUK ethics committee and the Duke University Institutional Review Board approved the study.

Data extraction was performed on each identified patient’s chart. Variables of interest included: age at initial diagnosis, gravidity and parity, insurance type, referral location, HIV status, initial procedure performed prior to transfer (if applicable), operations/procedures performed at CHUK, antibiotic administration (both prior and/or after admission), length of hospital stay at District Hospital, management at the referral hospital, and management at CHUK after transfer. Maternal deaths and causes of deaths were recorded. We incorporated the surgical component of the WHO ‘near miss’ criteria and focused on laparotomy and/or hysterectomy. Severe maternal morbidity (‘near miss’) was defined as requiring more than one laparotomy, and/or hysterectomy.

Statistical analysis was performed using STATA. Descriptive statistics, including patient characteristics, treatment prior to referral, and management at the referral center were calculated for the entire sample as well as the subsample of obstetrical patients. Differences in the primary outcome of severe maternal morbidity and mortality were assessed for statistical significance using the Fisher exact test.

## Results

Most (57 %) of patients were between 20 and 29 years old, although there was no significant difference in rate of severe morbidity or mortality by age group (Table 1). Patients were referred from all parts of the country, though more from the East province than others (Table 1). Geographic region of referral was not significantly associated with severe morbidity and mortality. Although about half of patients were primiparous, there was no significant association between parity and severe morbidity or mortality.

**Table 1:** Maternal demographics by composite maternal morbidity and mortality

	Obstetric patients (*N* = 117)	
Variable	Number (percentage)	Severe morbidity or mortality
Age		*p* = 0.34
< 20	11 (9 %)	7 (64 %)
20–29	67 (57 %)	53 (79 %)
30–39	33 (28 %)	25 (76 %)
> 39	6 (5 %)	5 (83 %)
Geographic region		*p* = 0.94
East province	53 (45 %)	42 (79 %)
Kigali	25 (21 %)	19 (76 %)
North province	18 (15 %)	14 (78 %)
South province	14 (12 %)	10 (71 %)
West province	7 (6 %)	5 (71 %)
Parity		*p* = 0.33
Nulliparous	11 (9 %)	7 (64 %)
Primiparous	51 (44 %)	38 (75 %)
Multiparous	55 (47 %)	45 (82 %)

Overall, 10 % of patients in the cohort were known to be HIV positive and 45 % had an unknown HIV status (Table 2). Patients with the default public insurance appeared to have a statistically significantly higher rate of severe morbidity and mortality (91 %) compared to those with better insurance (58 %) (Table 2). The predominant mode of delivery was cesarean section (81 % of patients). There was a statistically significant difference in outcome among methods of delivery, with cesarian delivery associated with a higher rate of severe morbidity and mortality (82 %) than abortion (50 %), and vaginal delivery (5 %) (Table 2).

**Table 2:** Mode of delivery and serostatus at the district hospital

Variable	Number (percentage)	Severe morbidity or mortality
Referred from district Hospital		
Yes	117 (100 %)	90 (77 %)
No	0 (0 %)	0 (N/A)
HIV positive		*p* = 0.16
Yes	12 (10 %)	7 (58 %)
No	52 (44 %)	39 (75 %)
Unknown	53 (45 %)	44 (83 %)
Insurance		*p* = 0.04
Public insurance	105 (90 %)	83 (80 %)
Other insurance, including private	12 (10 %)	7 (58 %)
Method of delivery		*p* = 0.04
Cesarean section	95 (81 %)	79 (83 %)
Abortion	14 (12 %)	7 (50 %)
Vaginal	8 (7 %)	5 (62 %)
Indication for cesarian section		*p* = 0.10
Unknown indication	43 (37 %)	34 (79 %)
Labor dystocia	21 (18 %)	18 (86 %)
Non-reassuring fetal status	12 (10 %)	10 (83 %)
Intrauterine fetal demise	9 (8 %)	8 (89 %)
Previous cesarian section	5 (4 %)	5 (100 %)
Breech presentation	2 (2 %)	2 (100 %)
Mitral stenosis	1 (1 %)	0 (0 %)
Twin pregnancy	1 (1 %)	1 (100 %)
Placenta abruption	1 (1 %)	1 (100 %)

Table 3 highlights initial the procedure performed at the District Hospital, procedure indications, and hospital course prior to referral. The majority of cases occurred after cesarean delivery (81 %), followed by septic abortion (12 %) and vaginal delivery (7 %). Among women who had cesarean deliveries, labor dystocia accounted for 18 %, previous cesarean accounted for 4 %, labor dystocia accounted for 18 %, and non-reassuring fetal status was attributed to 10 %. The indication was unknown in 37 %. Previous antibiotics were administered in 66 % of patients prior to transfer. The majority of patients had at least one surgery prior to transfer (79 %). Fifteen percent had two surgeries prior to transfer and, 1 % underwent three surgeries prior to admission to CHUK. Eighteen percent had no history of surgical procedure prior to transfer.

**Table 3:** Management and follow-up at district hospital

	Obstetric patients (*N* = 117)	
Variable	Number (percentage)	Severe morbidity or mortality
Procedure at district hospital		*p* = 0.19
Cesarean section	87 (74 %)	69 (79 %)
Septic abortion management	19 (16 %)	11 (58 %)
Laparotomy for IUFD	5 (4 %)	5 (100 %)
Laparotomy for uterine rupture repair	2 (2 %)	2 (100 %)
Laparotomy for ectopic pregnancy	1 (1 %)	1 (100 %)
Unknown	3 (3 %)	2 (67 %)
Surgeries prior to transfer		*p* = 0.01
0	22 (19 %)	13 (59 %)
1	77 (66 %)	59 (77 %)
2	17 (15 %)	17 (100 %)
3	1 (1 %)	1 (100 %)
Received antibiotics prior to transfer		*p* = 0.16
Yes	77 (66 %)	63 (82 %)
No	6 (5 %)	4 (67 %)
Unknown	34 (29 %)	23 (68 %)
Post-op day of transfer		*p* = 0.36
< 7 days	43 (37 %)	31 (72 %)
7–14 days	35 (30 %)	29 (83 %)
> 14 days	17 (15 %)	15 (88 %)
Unknown	22 (19 %)	15 (68 %)

Length of stay at the District Hospital prior to transfer was not associated with increased morbidity/mortality. Among patients who underwent surgery, 37 % were transferred within 7 days, 30 % within two weeks, 15 % were transferred more than two weeks following surgery. In 19 %, the interval between surgery and transfer was unknown.

The most common diagnosis upon arrival at the tertiary center was peritonitis (56 %). Deep surgical site infection, including fasciitis, complicated 17 % of cases and 15 % were diagnosed with endometritis. Hemoperitoneum and uterine dehiscence each occurred in 2 %. Tubo-ovarian abscess, retained placenta and foreign body each complicated 1 % of the population (Table 4). The most common initial procedure performed at the tertiary care center (CHUK) was exploratory laparotomy with washout (48 %). Twenty-eight percent of patients had non-surgical management and 22 % of patients experienced exploratory laparotomy with subtotal hysterectomy (Table 4).

**Table 4:** Diagnosis and initial management at tertiary center (CHUK)

	Obstetric patients (*N* = 117)	
Variable	Number (percentage)	Severe morbidity or mortality
Diagnosis at tertiary center		*p* < 0.01
Peritonitis	66 (56 %)	62 (94 %)
Wound infection	20 (17 %)	12 (60 %)
Endometritis	18 (15 %)	7 (39 %)
Parietal infection	4 (3 %)	2 (50 %)
Hemoperitoneum	2 (2 %)	2 (100 %)
Necrotizing fasciitis	2 (2 %)	1 (50 %)
Uterine dehiscence	2 (2 %)	2 (100 %)
Foreign body	1 (1 %)	1 (100 %)
Retained placenta	1 (1 %)	0 (0 %)
Tubo-ovarian abscess	1 (1 %)	1 (100 %)
Initial management at tertiary center		*p* < 0.01
Exploratory laparotomy with washout	56 (48 %)	51 (91 %)
Non-surgical management	33 (28 %)	12 (36 %)
Exploratory laparotomy with subtotal hysterectomy	26 (22 %)	26 (100 %)
Other surgical management	2 (1 %)	0 (0 %)
Debridement only	1 (1 %)	1 (100 %)

The overall maternal mortality among patients admitted with severe post-partum infections was 5 % (6 deaths). Sepsis was the cause of death for the majority of patients (67 %). The primary procedure performed for all patients was cesarean delivery.

## Discussion and conclusion

The aim of this study was to measure severe morbidity and mortality associated with post-partum infection in the largest tertiary care center in Rwanda. In Sub-Saharan Africa, sepsis is attributed to 9.7 % of maternal deaths [6]. Maternal mortality secondary to infection was 5 % in our study. Sepsis was the direct cause of death in 4 of the 6 cases.

All 6 maternal deaths occurred following cesarean sections. In Rwanda, the overall cesarean delivery rate is 15 %[4]. The rate varies by level of care; health center and district hospital cesarean delivery rates are lower than tertiary care centers. At the level of the district hospital, cesarean delivery rates increased from 36 % in 2010 to 45 % in 2011. These rates parallel reductions in maternal and infant deaths and are considered partially responsible for Rwanda’s success in reaching MDGs for maternal and infant death [10]. The increase in access to cesarean deliveries is a component of maternal mortality reduction, however, the association between high cesarean delivery rates and infection related maternal morbidity and mortality noted in this tertiary care setting is concerning. Cesarean deliveries are considered essential components of emergency obstetric service and some centers in Sub-Saharan Africa have demonstrated a balance between increasing access to cesarean deliveries in tertiary settings without significant increases in surgical site infections [11]. These data demonstrate the potential risks associated with high cesarean delivery rates. These procedures may trigger the cascade leading to severe maternal morbidity and mortality from infectious complications [12].

In general, patients underwent hysterectomy based on providers’ clinical impression at the time of laparotomy. The decision to debride necrotic uterine tissue or proceed with hysterectomy was often based on patient’s parity, extent of necrosis, prior procedures, and overall clinical status. At the time of this study, no standard practice or protocols guided management in our setting.

The high numbers of peritonitis cases is concerning. The reasons are unclear. They may reflect a lack of surgical experience, sterile conditions, lack of materials, antibiotic prophylaxis or the increasing prevalence of multidrug resistant organisms in Rwanda [13, 14].

Almost half of patients in this study were transferred from the Eastern province of Rwanda, as expected given transfer patterns. Previous work in our center demonstrated a high rate of near misses and maternal mortality from this region of the country [8]. These findings may reflect the proximity of CHUK to this region. CHUK is the nearest tertiary care hospital to this province. Furthermore, fewer transfers originate from the south and Western province, largely because those patients are transferred to the University Teaching Hospital of Butare (CHUB). Additional work may further elucidate reasons for high rates of maternal morbidity and mortality originating from the Eastern region of the country.

The primary limitation of this study is the retrospective design. Initial assessment information was based on the transfer sheets. These communications often lacked basic information about the patient’s hospital course prior to transfer to the tertiary care center (CHUK). In many cases the accepting provider had to call the referring institution to obtain additional information. Despite this approach, some information was still not obtained by phone. Therefore, many of the risk factors leading to near misses and mortalities remain poorly understood. Despite efforts to extract additional information from chart reviews, some data are still missing.

CHUK is the largest public tertiary hospital in Rwanda. These findings may not reflect outcomes at the level of the district hospital or community health center.

The available data did not allow for determination of the root causes of near misses and maternal mortalities. Prospective audits and surveillance of severe maternal morbidity and mortality are ongoing. The evaluation of these events for preventability remains a high priority for the CHUK obstetrics and gynecology department.

This study provides an important picture of the problems encountered in the largest public tertiary care center in Rwanda. These findings highlight the need to establish consistent management approaches to patients with peritonitis and surgical site infections at CHUK. Since the completion of this study, CHUK established an ongoing, prospective database of all admissions for peritonitis. These results enhance our understanding of a significant, potentially preventable, cause of near miss and maternal mortality in Rwanda.

## Abbreviations

CHUB, University Teaching Hospital of Butare; CHUK, University Teaching Hospital of Kigali; MDG, Millennium Development Goal; MMR, Maternal Mortality Ratio

## References

[1] Hogan MC, Foreman KJ, Naghavi M, Ahn SY, Wang MR, Makela SM, Lopez AD, Lozano R, Murray CJL (2010). Maternal mortality for 181 countries, 1980–2008: a systematic analysis of progress towards Millennium Development Goal 5. Lancet (British edition).

[2] United Nations Sustainable Development Goals. http://www.un.org/sustainabledevelopment/sustainable-development-goals/ (Accessed on April 15, 2016).

[3] Health Mo (2015). Rwanda Demographic and Health Survey 2014–15: key indicators.

[4] Ministry of Health R. Rwanda Ministry of Health Annual Health Statistics Booklet 2011.

[5] Brubaker SG (2014). Patterns of use and predictors of receipt of antibiotics in women undergoing cesarean delivery. Obstet Gynecol.

[6] Burchett HE, Mayhew SH (2009). Maternal mortality in low-income countries: what interventions have been evaluated and how should the evidence base be developed further?. Int J Gynaecol Obstet.

[7] Jackson RRS, Decesare J, Williams B, Hill W (2015). Maternal morbidity and mortality in the University Teaching Hospital in Kigali, Rwanda. Rwanda Med J.

[8] Rulisa S, Umuziranenge I, Small M, van Roosmalen J (2015). Maternal near miss and mortality in a tertiary care hospital in Rwanda. BMC Pregnancy Childbirth.

[9] WHO. Evaluating the quality of care for severe pregnancy complications. The WHO near miss approach for maternal health. World Health Organization; 2011.

[10] National Institute of Statistics of Rwanda, Ministry of Health, MEASURE DHS (Program) (2011). Rwanda demographic and health survey 2010: final report.

[11] Chu K, Maine R, Trelles M (2015). Cesarean section surgical site infections in sub-Saharan Africa: a multi-country study from Medecins Sans Frontieres. World J Surg.

[12] Litorp H, Kidanto HL, Roost M, Abeid M, Nystrom L, Essen B (2014). Maternal near-miss and death and their association with caesarean section complications: a cross-sectional study at a university hospital and a regional hospital in Tanzania. BMC Pregnancy Childbirth.

[13] Mivumbi VN, Little SE, Rulisa S, Greenberg JA (2014). Prophylactic ampicillin versus cefazolin for the prevention of post-cesarean infectious morbidity in Rwanda. Int J Gynaecol Obstet.

[14] Ntirenganya C, Manzi O, Muvunyi CM, Ogbuagu O (2015). High prevalence of antimicrobial resistance among common bacterial isolates in a tertiary healthcare facility in Rwanda. Am J Trop Med Hyg.

