# Erratum to: Maternal near miss and mortality due to postpartum infection: a cross-sectional analysis from Rwanda

**DOI:** 10.1186/s12884-017-1368-7

**Published:** 2017-06-06

**Authors:** Denis Rwabizi, Stephen Rulisa, Aidan Findlater, Maria Small

**Affiliations:** 1 Department of Obstetrics and Gynecology, University of Rwanda, BP 655 Kigali, Rwanda; 2 Department of Obstetrics and Gynecology, University Teaching Hospital of Kigali, Kigali, Rwanda; 3 University of Western Ontario, London, ON Canada; 4 Department of Obstetrics and Gynecology, Duke University, Durham, NC USA

## Erratum

Upon publication of the original article [1], it was noticed that the author’s name “Aidan Findlater” was incorrectly given as “Findlater Aidan”. This has now been acknowledged and corrected in this erratum.

## References

[1] Rwabizi D, Rulisa S, Aidan F, Small M (2016). Maternal near miss and mortality due to postpartum infection: a cross-sectional analysis from Rwanda. BMC Pregnancy and Childbirth.

